# Food Insecurity Is Associated with Increased Risk of Obesity in US College Students

**DOI:** 10.1093/cdn/nzaa120

**Published:** 2020-07-15

**Authors:** Aseel El Zein, Sarah E Colby, Wenjun Zhou, Karla P Shelnutt, Geoffrey W Greene, Tanya M Horacek, Melissa D Olfert, Anne E Mathews

**Affiliations:** Food Science and Human Nutrition Department, University of Florida, Gainesville, FL, USA; Department of Nutrition, University of Tennessee, Knoxville, TN, USA; Department of Business Analytics and Statistics, University of Tennessee, Knoxville, TN, USA; Department of Family, Youth, and Community Sciences, University of Florida, Gainesville, FL, USA; Department of Nutrition and Food Sciences, University of Rhode Island, Kingston, RI, USA; Nutrition Science and Dietetics, Syracuse University, Syracuse, NY, USA; Division of Animal and Nutritional Sciences, Davis College of Agriculture, Natural Resources and Design, West Virginia University, Morgantown, WV, USA; Food Science and Human Nutrition Department, University of Florida, Gainesville, FL, USA

**Keywords:** food insecurity, obesity, college students, dietary intake, meal plan, sex

## Abstract

**Background:**

Food insecurity affects millions of Americans and college students are especially vulnerable. Little is known about the relation of food insecurity with weight status and dietary intake during this critical phase of emerging adulthood.

**Objectives:**

We aimed to examine the sex-specific associations of food insecurity with obesity and dietary intake among college students. The study also explored these associations by meal plan (MP) enrollment.

**Methods:**

This cross-sectional study included 683 second-year students at 8 universities in the United States. Food security status and dietary intake were assessed using the USDA Adult Food Security Survey and the Dietary Screener Questionnaire, respectively. On-site anthropometrics were measured by researchers.

**Results:**

The prevalence of food insecurity at the universities ranged from 19.0% to 34.1% with a mean of 25.4% for the entire sample. Compared with high food security, marginal food security and food insecurity were associated with 3.16 (95% CI: 1.55, 6.46) and 5.13 (95% CI: 2.63, 10.00) times increased odds of obesity, respectively, exhibiting a dose–response relation. Food insecurity remained a significant predictor of obesity among both sexes after adjusting for sociodemographic variables. Food-insecure (FI) students had a significantly lower intake of fruits and vegetables and higher intake of added sugars than food-secure (FS) students. Obesity rate and added sugars consumption were higher among FI students with MPs than among FI students lacking MPs and FS students regardless of MP status. Among students with MPs, FS students had a higher intake of fruits and vegetables than FI students.

**Conclusions:**

Food insecurity was associated with obesity and poor dietary intake among both sexes. Although MP subsidies may be a reasoned approach to combat food insecurity, it should be coupled with efforts to assist students in making healthy food choices.

Registered at clinicaltrials.gov as NCT02941497.

## Teaser

Food insecurity is associated with obesity and poor diet habits among male and female college students. Obesity rate and added sugars intake were highest among food-insecure students with meal plans.

## Introduction

Reducing obesity in college students may be possible if specific economic stressors are identified and addressed by applicable social policies. Prior research has identified food insecurity, defined as limited access to adequate and safe foods (1), as one of the stressors linked to obesity and associated comorbidities in adults (1–3). Indicators of food insecurity include being unable to purchase nutritionally balanced foods, having anxiety that food will run out, and skipping meals because of limited food supply (3). More detrimental indicators of food insecurity include going an entire day without eating owing to financial constraints. Yet, food insecurity is described as a “managed process,” suggesting that individuals will work diligently to avoid hunger (4). Although food-insecure (FI) individuals may react differently to having limited finances for nutritionally balanced foods, it is common for dietary quality to decline when food is scarce (5). This is supported by the inverse relation between energy density and cost of foods (5, 6). Indeed, according to the insurance hypothesis posited by Nettle et al. (7), humans possess decision-making mechanisms that often cause them to increase their energy intake above their energy expenditure when they receive cues that access to food is limited.

The association of food insecurity with obesity was first proposed in 1995 by Dietz (8) who suggested that “food choices or physiologic adaptations to episodic food shortages could cause increased body fat.” In addition to dependence on low-cost and palatable foods to stretch food dollars, FI individuals may have limited knowledge, time, and resources to engage in healthy eating habits, which can result in increased consumption of saturated fats, added sugars, and refined grains (9, 10). It has also been hypothesized that FI individuals alternate between times of adequate food availability and food scarcity (11). During times of unpredictable food supply, FI individuals engage in a poverty-related food restriction, promoting dependence on inexpensive and energy-dense foods. When food is available through monthly paychecks, food assistance, or social invitations, the same individuals may engage in overeating behaviors resulting in a physiological shift toward energy efficiency and increased storage of fat (9). Repeated episodes of these behaviors may trigger a vicious cycle of eating-related stress and disordered eating behaviors (12). Thus, minimizing these behaviors through consistent access to food may have implications for the prevention and management of poor dietary intake, obesity, and associated comorbidities.

Despite a growing body of evidence to indicate a link between food insecurity and obesity, studies among college students have yielded mixed results (2, 13). Prior research (13, 14) examining the obesity–food insecurity connection among college students has been limited to single institutions and relied on self-reported body weight rather than measured body weight (15), which may have masked the association between food insecurity and obesity (16). Also, there appears to be a sex disparity in the association of food insecurity and obesity (17). For example, in a nationally representative sample of 9479 nonstudent respondents, food insecurity was associated with obesity among women, but not among men (11, 18). Food intake and preferences generally differ between men and women, which can be related to social desirability or biological factors (19, 20). Whether this sex difference in the association between food insecurity and obesity exists in the college setting and is pertinent to dietary intake remains unknown. Unique to this phase of life, many college students have at least some access to food through campus meal plans (MPs), and reduced-cost MPs have been proposed to address food insecurity. However, to our knowledge no study to date has examined the differences in dietary intake and weight status of food-secure (FS) and FI students enrolled in MPs compared with those who are not. These findings and inconsistencies necessitate further research to clarify the association between food insecurity and obesity using objective measures among this population. Further, examining the intersection of MP enrollment with food insecurity, diet, and weight status could lead to interventions targeted at improving food security status and preventing obesity in college students.

To investigate these dynamics, we *1*) examined the association between food security status and odds of obesity [defined as BMI (in kg/m^2^) ≥30] stratified by sex; *2*) compared the differences in anthropometric and dietary variables by food security status, and, lastly, *3*) explored the difference in dietary variables and weight status by MP enrollment among FS and FI students.

## Methods

### Study overview

This cross-sectional study was a secondary analysis of data collected at 8 universities (University of Florida, Auburn University, South Dakota State University, University of Maine, West Virginia University, Kansas State University, Syracuse University, and University of Tennessee) across the United States under a USDA-funded research project called Get Fruved (NCT02941497). Data collection took place during April 2017 on a sample of second-year students who were invited for an in-person assessment. Participants completed a battery of health-related questionnaires delivered through a web-based format and had their anthropometrics measured by trained researchers. The University of Tennessee Institutional Review Board reviewed and provided ethical approval for all study activities at West Virginia University, South Dakota State University, University of Maine, Syracuse University, and the University of Tennessee. The Institutional Review Boards at the University of Florida, Auburn University, and Kansas State University reviewed and approved the study for their respective campuses, and written informed consent was obtained from all participants before completing the questionnaire and assessment procedures.

### Study population

In accordance with the design of the parent study, participants were eligible based on eating <2 cup equivalents (CE) of fruit and/or <3 CE of vegetables per day as measured by the National Cancer Institute's (NCI's) 9-item all-day screener (21) and having 1 additional self-reported risk factor for poor health behaviors. These included identifying as a first-generation college student, having an overweight or obese parent, identifying as racial/ethnic minority, having a BMI ≥25, or reporting low family affluence. Eligibility criteria were selected based on the purpose of the parent study, which was to promote healthy lifestyle behaviors among college students.

### Food security measurement

Food security was measured using the 10-item validated Adult Food Security Survey (AFSS) developed by the USDA (22). The questions assess the severity of food insecurity and range from concerns that food would run out before participants had money to purchase more to whether they did not eat for a whole day owing to financial constraints. The number of affirmative responses was calculated to generate a food security score from 0 to 10. Responses were then grouped into 4 categories: high food security, indicating no food access problems (raw score of 0); marginal food security, indicating anxiety over food supply (raw score of 1–2); low food security, indicating reduced quality and variety of the diet (raw score of 3–5); and very low food security, highlighting the presence of disrupted eating patterns and reduced food intake, customarily designated as “food insecure with hunger” (raw score of 6–10). According to the USDA definitions, these categories were further collapsed into either FS (high and marginal food security status) or FI (low or very low food security status). All scoring procedures were in line with the Guide to Measuring Food Security (23) and the USDA's definitions of food security (24).

### Anthropometric measurements

Anthropometric assessments for study participants were conducted by trained research assistants who had undergone interrater reliability testing, using standard techniques and equipment. Standing height and weight were measured using portable stadiometers and digital floor scales to the nearest 0.1 cm and 0.1 kg, respectively. Participants were dressed in minimal clothes without shoes. BMI was calculated as kg/m^2^, and obesity was defined as BMI ≥30. Waist circumference (WC) was measured to the nearest 0.1 cm using a Gulick tape measure (North Coast Medical) at the midpoint between the bottom of the rib cage and the top of the iliac crest, and hip circumference (HC) at the largest circumference of the hips. Both WC and HC were recorded to the nearest 0.1 cm. Neck circumference (NC) was also measured as it has been associated with obesity-related conditions and chronic diseases (25). Measurement was taken immediately above the laryngeal prominence (Adam's apple) while participants were standing with eyes facing forward. All measures were taken twice and the mean of the 2 values was reported.

### Dietary intake

The NCI's 26-item validated Dietary Screener Questionnaire was used to assess the frequency of intake in the past month for selected foods/drinks. Participants were asked to consider meals and snacks eaten at home, work, school, restaurants, and any other locations. Responses were converted to estimated daily intake values using scoring algorithms provided by the NCI (26). These included daily estimates of fruits and vegetables (CE/d), added sugars from sugar-sweetened beverages (SSBs) (tsp/d), total added sugars from beverage and nonbeverage sources (tsp/d), fiber (g/d), whole grains [ounce (oz)/d], dairy products (CE/d), and calcium (mg/d).

### Sociodemographic characteristics

The remaining variables captured demographic and economic status. Demographics included sex (male/female), age (18–19/20–21 y), marital status, housing (on-campus/off-campus), race/ethnicity (non-Hispanic black/non-Hispanic white/Hispanic-Latino/other or multiracial), and parental education (high school or lower/some college or higher). Economic variables included Pell Grant receipt (receives Pell Grant/does not receive Pell Grant), employment (employed/unemployed), and MP (has an MP/does not have an MP).

### Statistical analyses

Descriptive statistics were used to report the prevalence of food insecurity, weight status, and the distribution of other participant characteristics, either as frequencies and proportions for categorical variables or as means with SEs for continuous variables. Using logistic regression models, crude and adjusted ORs were reported for associations between food insecurity and sociodemographic characteristics. The outcome in these models was food insecurity, which was recoded into 2 categories: 0 = FS compared with 1 = FI.

To analyze the bivariate association of anthropometric and dietary variables with food insecurity, both in aggregate and by gender, *t* tests or Mann–Whitney *U* tests (when distributions were nonnormally distributed) were used. Adjusted and unadjusted multiple logistic regression models were conducted to examine the association between food security status and obesity. This analysis was done using a 2-level coding and 3-level coding of food security status. In the adjusted analysis, models were performed including all sociodemographic variables that were significant in the bivariate analysis or hypothesized to influence food security status and obesity. Results from the logistic regression models were expressed as ORs with 95% CIs.

Finally, to examine the statistical differences in anthropometric measurements and dietary intake of FS and FI students with and without MPs, 1-factor ANOVA (or a chi-square test for a categorical variable) was performed among the following 4 groups: FS with MP, FS without MP, FI with MP, and FI without MP. When ANOVA indicated significant differences (*P* < 0.05), the Tukey test was used for post hoc comparisons to identify the groups that differed.

For statistical tests, a *P* value < 0.05 was considered significant. All data analyses were conducted using IBM SPSS Statistics for Windows, version 24.

## Results

Data were obtained from 683 college students. The majority were 20 y old (61.2%), female (69.6%), and non-Hispanic white (47.8%). Around half (52.4%) of the students resided off-campus, almost three-fifths (59.6%) were enrolled in a MP, and almost two-thirds were employed either part time or full time (63.0%). The distribution of student enrollment was as follows: University of Florida, 32.2%; Syracuse University, 14.7%; University of Maine, 14.1%; University of Tennessee, 13.1%; Kansas State University, 10.7%; West Virginia University, 6.6%; South Dakota State University, 4.2%; and Auburn University, 4.2%.

Using simple logistic regression analysis, the correlates of food insecurity were housing, employment, Pell Grant, and MP status (**Table 1**). Results from the multiple logistic regression analyses showed that students who lived off-campus were more likely to be FI than those who resided on-campus (OR: 1.99; 95% CI: 1.33, 2.98), and those who did not have an MP were more likely to be FI than those who had an MP (OR: 1.37; 95% CI: 1.22, 2.52). In addition, Pell Grant recipients had higher odds of food insecurity than nonrecipients (OR: 1.95; 95% CI: 1.35, 2.82), and employed students had higher odds of food insecurity than unemployed students (OR: 1.60; 95% CI: 1.08, 2.37).

**TABLE 1:** Sociodemographic characteristics associated with food insecurity in second-year college students, USA, 2017^1^

Sociodemographic characteristic	Total (*n* = 683)	FS (*n* = 509)	FI (*n* = 174)			Adjusted OR^3^	
	*n* (%)	*n* (%)	*n* (%)	Crude OR^2^	95% CI		95% CI
Age, y							
18–19	241 (35.6)	177 (35.1)	63 (36.8)	1.00	Ref.	1.00	Ref.
20–21	436 (64.4)	327 (64.9)	108 (63.2)	0.92	0.64, 1.33	0.96	0.65, 1.41
Sex							
Male	205 (30.4)	149 (29.6)	56 (32.6)	1.00	Ref.	1.00	Ref.
Female	470 (69.6)	354 (70.4)	116 (67.4)	1.14	0.79, 1.66	0.95	0.47, 1.06
Race/ethnicity							
Non-Hispanic white	321 (47.8)	248 (49.6)	71 (42.0)	1.00	Ref.	1.00	
Non-Hispanic black	89 (13.3)	59 (11.8)	30 (17.8)	0.73	0.23, 2.32	0.45	0.08, 2.33
Hispanic/Latino	138 (20.6)	98 (19.6)	40 (23.7)	0.59	0.19, 1.80	0.47	0.09, 2.35
Other/multiracial	123 (18.3)	95 (19.0)	28 (16.6)	0.53	0.16, 1.71	0.33	0.06, 1.72
Marital status							
Single	394 (59.2)	295 (59.0)	99 (59.6)	1.00	Ref.	1.00	Ref.
In a relationship	272 (40.8)	205 (41.0)	67 (40.4)	0.97	0.68, 1.39	1.16	0.76, 1.77
Pell Grant status							
No	264 (39.9)	317 (64.3)	81 (47.9)	1.00	Ref.	Ref.	
Yes	398 (60.1)	176 (35.7)	88 (52.1)	1.95**	1.37, 2.78	1.95**	1.35, 2.82
Housing							
On-campus	317 (47.6)	258 (52.1)	59 (34.5)	1.00	Ref.	1.00	
Off-campus	349 (52.4)	237 (47.9)	112 (65.5)	2.06**	1.44, 2.96	1.99**	1.33, 2.98
Meal plan							
Yes	399 (59.6)	313 (63.1)	86 (49.7)	1.00	Ref.	1.00	Ref.
No	270 (40.4)	183 (36.9)	87 (50.3)	1.73**	1.22, 2.45	1.37**	1.22, 2.52
Employment status							
Unemployed	249 (37.0)	199 (39.6)	121 (70.8)	1.00	Ref.	1.00	Ref.
Employed (part/full time)	424 (63.0)	303 (60.4)	50 (29.2)	1.58*	1.12, 2.31	1.60*	1.08, 2.37
Mother's education							
High school or less	299 (44.7)	153 (44.1)	61 (51.7)	1.00	Ref.	1.00	Ref.
Some college or higher	370 (55.3)	194 (55.9)	57 (48.3)	0.73	0.48, 1.12	0.84	0.45, 1.46
Father's education							
High school or less	338 (52.1)	190 (56.5)	57 (58.3)	1.00	Ref.	1.00	Ref.
Some college or higher	311 (47.9)	146 (43.5)	48 (41.7)	0.93	0.60, 1.43	0.81	0.49, 1.32

1 ^*,**^Statistically significant difference from reference category: *P < 0.05, **P < 0.01. FI, food-insecure; FS, food-secure.

2 Crude OR refers to unadjusted OR of food insecurity among the study sample.

3 Adjusted OR refers to OR of food insecurity after adjusting for all other sociodemographic variables.

Results of the food security survey showed that 25.4% (95% CI: 22.2%, 28.9%) of the respondents were FI (**Table 2**). These include students with low food security status (14.3%; 95% CI: 11.8%, 17.2%) and very low food security status (11.1%; 95% CI: 8.9%, 13.7%). An additional 22.3% (95% CI: 19.2%, 25.6%) were at risk of becoming FI and these fell in the marginal food security category. The highest prevalence of food insecurity was reported among students from University of Tennessee (33.8%; 95% CI: 24.3%, 45.0%), with Kansas State University having the second-highest prevalence (32.0%; 95% CI: 21.1%, 42.9%); these were followed by University of Florida (24.8%; 95% CI: 19.1%, 31.1%), West Virginia University (24.4%; 95% CI: 12.9%, 39.5%), Auburn University (24.1%; 95% CI: 10.3%, 43.5%), Syracuse University (23.0%; 95% CI: 15.2%, 32.5%), South Dakota State University (20.6%; 95% CI: 8.0%, 39.7%), and University of Maine (19.0%; 95% CI: 11.6%, 28.3%). The prevalence of overweight and obesity among the study sample was 30.3% (95% CI: 26.8%, 34.0%) and 10.5% (95% CI: 8.3%, 13.1%), respectively.

**TABLE 2:** Food security and weight status of second-year college students, USA, 2017^1^

	Food security status				Weight status			
University, *n*	High food security	Marginal food security	Low food security	Very low food security	Underweight	Normal weight	Overweight	Obese
University of Florida, 221	121 (54.8)	45 (20.4)	35 (15.8)	20 (9.0)	9 (4.2)	134 (62.6)	59 (27.6)	12 (5.6)
Syracuse University, 100	55 (55.0)	22 (22.0)	14 (14.0)	9 (9.0)	5 (5.1)	64 (64.6)	22 (22.2)	8 (8.1)
University of Maine, 95	53 (55.8)	24 (25.3)	9 (9.5)	9 (9.5)	3 (3.3)	46 (50.0)	30 (32.6)	13 (14.1)
University of Tennessee, 89	41 (46.1)	18 (20.2)	15 (16.9)	15 (16.9)	3 (3.5)	39 (45.3)	29 (33.7)	15 (17.4)
Kansas State University, 75	38 (50.7)	13 (17.3)	13 (17.3)	11 (14.7)	0 (0.0)	32 (47.1)	32 (47.1)	4 (5.9)
West Virginia University, 45	20 (44.4)	14 (31.1)	4 (8.9)	7 (15.6)	1 (2.4)	22 (52.4)	11 (26.2)	8 (19.0)
Auburn University, 29	15 (51.7)	7 (24.1)	3 (10.3)	4 (13.8)	2 (6.9)	18 (62.1)	5 (17.2)	4 (13.8)
South Dakota State University, 29	14 (48.3)	9 (31.0)	5 (17.2)	1 (3.4)	0 (0.0)	10 (38.5)	11 (42.3)	5 (19.2)
Total	357 (52.3)	152 (22.3)	98 (14.3)	76 (11.1)	23 (3.5)	365 (55.6)	199 (30.3)	69 (10.5)

1 *n* = 683. Values are *n* (%) unless otherwise indicated.

Significant associations were noted when comparing FS with FI students on anthropometric variables (**Table 3**). In the overall analytic sample, FI students had a significantly higher BMI than FS students (26.0 ± 0.4 compared with 24.2 ± 0.1, *P* = 0.001). Female FI students had a significantly higher WC (80.2 ± 1.2 compared with 76.5 ± 0.4 cm, *P* = 0.009), HC (104.0 ± 1.0 compared with 99.0 ± 0.4 cm), and BMI (26.0 ± 0.5 compared with 23.9 ± 0.2) than their FS counterparts. There were no anthropometric differences in male students.

**TABLE 3:** Anthropometric measurements and dietary variables by food security status of second-year college students, USA, 2017^1^

		Total sample			Men			Women		
	Total (*n* = 683)	FS (*n* = 509)	FI (*n* = 174)	*P* value	FS (*n* = 150)	FI (*n* = 56)	*P* value	FS (*n* = 359)	FI (*n* = 118)	*P* value
Anthropometrics										
Weight, kg	69.6 ± 0.5	68.6 ± 0.5	71.2 ± 1.3	0.022	77.9 ± 1.0	77.3 ± 2.2	0.781	65.0 ± 0.6	70.0 ± 1.6	0.001
Height, cm	165.0 ± 0.3	168.2 ± 0.3	167.2 ± 0.6	0.178	177.0 ± 0.5	174.8 ± 1.0	0.058	164.6 ± 0.3	163.8 ± 0.6	0.256
BMI, kg/m^2^	24.6 ± 0.1	24.2 ± 0.1	26.0 ± 0.4	0.001	24.8 ± 0.3	25.4 ± 0.7	0.437	23.9 ± 0.2	26.0 ± 0.5	0.001
WC, cm	79.3 ± 0.4	78.6 ± 0.4	81.4 ± 0.4	0.011	83.5 ± 0.7	85.6 ± 1.7	0.532	76.5 ± 0.4	80.2 ± 1.2	0.009
HC, cm	101.0 ± 0.3	100.4 ± 0.3	102.6 ± 0.8	0.016	101.9 ± 0.5	100.4 ± 1.4	0.269	99.0 ± 0.4	104.0 ± 1.0	<0.001
NC, cm	33.9 ± 0.1	33.7 ± 1.4	34.2 ± 0.2	0.073	37.5 ± 0.1	37.3 ± 0.3	0.621	32.2 ± 0.1	32.8 ± 0.2	0.105
Dietary intake										
FV, CE/d	2.0 ± 0.02	2.2 ± 0.03	1.6 ± 0.05	0.001	2.3 ± 0.07	1.8 ± 0.07	0.002	2.0 ± 0.04	1.6 ± 0.06	0.027
Dairy, CE/d	1.3 ± 0.03	1.3 ± 0.03	1.3 ± 0.06	0.921	1.8 ± 0.09	1.6 ± 0.1	0.295	1.1 ± 0.03	1.2 ± 0.07	0.375
Calcium, mg/d	808 ± 18.0	814 ± 22.2	792 ± 28.7	0.590	1070 ± 52.8	943 ± 61.2	0.178	705 ± 19.7	717 ± 28.1	0.776
Whole grains, oz/d	0.6 ± 0.03	0.6 ± 0.03	0.7 ± 1.0	0.105	0.7 ± 0.07	0.8 ± 1.0	0.668	0.5 ± 0.03	0.6 ± 1.0	0.100
Fiber, g/d	13.2 ± 0.1	13.3 ± 0.2	12.9 ± 0.2	0.262	15.4 ± 0.3	14.1 ± 0.4	0.061	12.4 ± 0.2	12.3 ± 0.3	0.693
Added sugars, tsp/d	12.4 ± 0.2	11.5 ± 0.2	14.0 ± 0.1	0.001	14.2 ± 0.5	16.5 ± 0.8	0.009	10.7 ± 0.2	12.4 ± 0.6	0.019
Sugar from SSBs, tsp/d	4.7 ± 0.1	4.5 ± 0.2	6.4 ± 0.5	<0.001	5.4 ± 4.6	8.3 ± 6.4	0.001	3.6 ± 0.2	5.5 ± 0.6	0.005

^1^Values are means ± SEs. CE, cup-equivalent; FI, food insecure; FS, food secure; FV, fruits and vegetables; HC, hip circumference; NC, neck circumference; SSB, sugar-sweetened beverage; WC, waist circumference.

Differences in food consumption of FS compared with FI students were also observed (Table 3). Among the entire analytic sample, FI students had a significantly lower intake of fruits and vegetables (1.6 ± 0.05 compared with 2.2 ± 0.03 CE/d, *P* = 0.001) than FS students. On the other hand, FI students had higher intakes of sugar from SSBs (6.4 ± 0.5 compared with 4.5 ± 0.2 tsp/d, *P* < 0.001) and total added sugars than FS students (14.0 ± 0.5 compared with 11.5 ± 0.2 tsp/d, *P *= 0.002). These dietary differences were consistent across both sexes.

**FIGURE 1:**
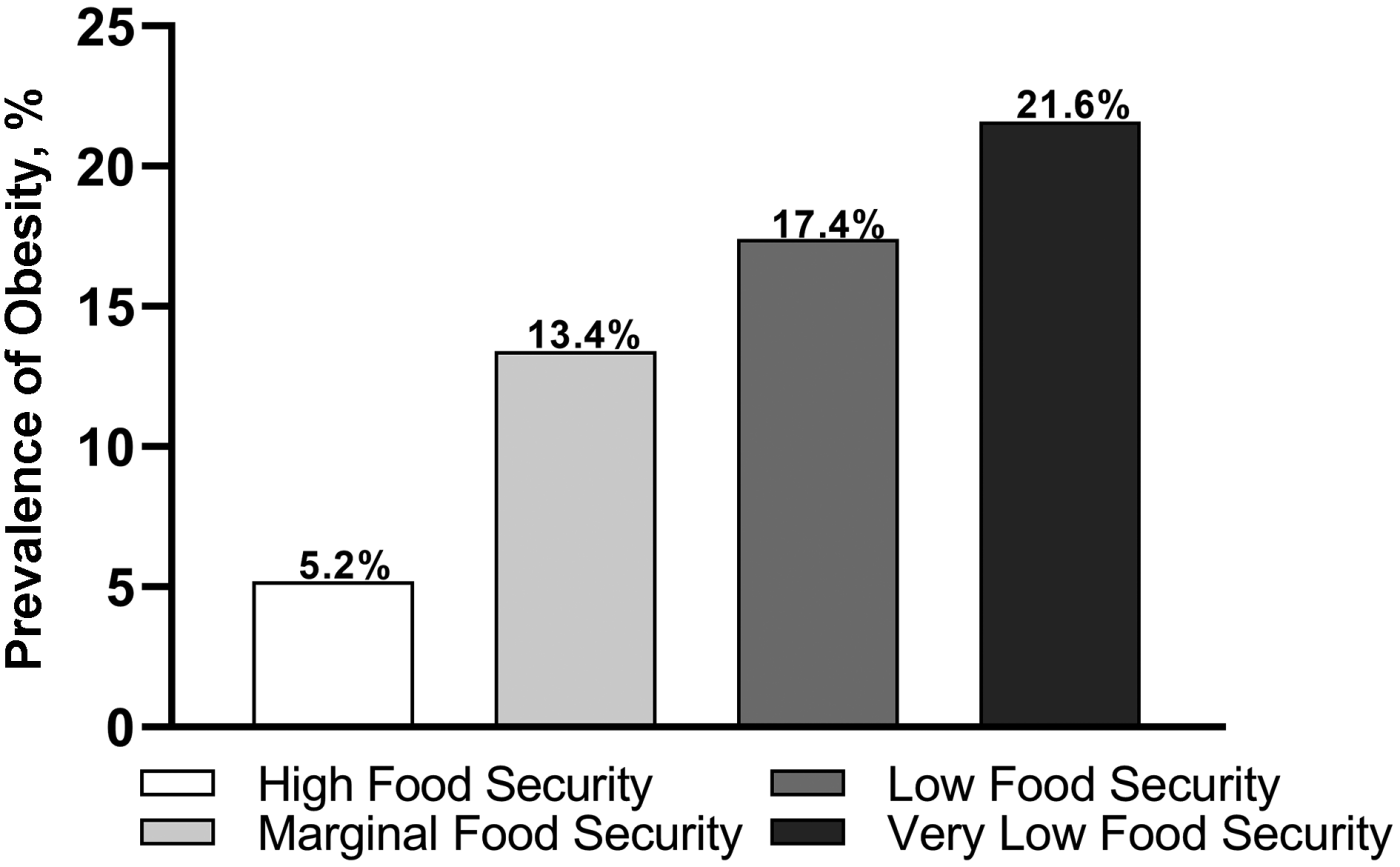
Prevalence of obesity (BMI ≥30 kg/m^2^) by food security status among second-year college students (*n* = 683), 2017. The prevalence of obesity increases with worsening food security status [χ^2^ (3) = 26.11, *P* < 0.001].

The trend in obesity increased with the degree of food insecurity (**Figure 1**). In logistic regression analysis, compared with students with high food security, marginal food security and food insecurity were associated with 3.16 (95% CI: 1.55, 6.46) and 5.13 (95% CI: 2.63, 10.00) times increased odds of obesity, respectively, exhibiting a dose–response relation. Food insecurity remained a significant predictor of obesity among men (OR: 3.84; CI: 1.47, 10.02) and women (OR: 2.88; 95% CI: 1.46, 5.71) after adjustment for potential demographic confounders, including university, age, marital status, employment, race/ethnicity, and MP enrollment (**Table 4**).

**TABLE 4:** Association of obesity with food security status among second-year college students, USA, 2017^1^

	Total sample (*n* = 683)		Men (*n* = 206)		Women (*n* = 477)	
Food security status	OR (95% CI)	*P* value	OR (95% CI)	*P* value	OR (95% CI)	*P* value
2-Level coding						
Unadjusted^2^						
FS	Ref.		Ref.		Ref.	
FI	2.92 (1.75, 4.87)	<0.001	3.25 (1.31, 8.00)	0.011	2.78 (1.49, 5.17)	0.001
Adjusted^3^						
FS	Ref.		Ref.		Ref.	
FI	3.07 (1.77, 5.31)	<0.001	3.84 (1.47, 10.02)	0.005	2.88 (1.46, 5.71)	0.004
3-Level coding						
Unadjusted^2^						
Highly FS	Ref.		Ref.		Ref.	
Marginally FS	2.83 (1.43, 5.57)	0.003	1.16 (0.29, 4.63)	0.799	4.06 (1.77, 9.02)	0.001
FI	4.37 (2.37, 8.06)	<0.001	3.40 (1.27, 9.08)	0.015	5.16 (2.34, 11.37)	<0.001
Adjusted^3^						
Highly FS	Ref.		Ref.		Ref.	
Marginally FS	3.16 (1.55, 6.46)	0.004	1.31 (0.29, 5.85)	0.717	4.39 (1.83, 10.51)	0.001
FI	5.13 (2.63, 10.00)	<0.001	4.68 (1.57, 13.92)	0.005	5.80 (2.43, 13.82)	<0.001

1 FI, food-insecure; FS, food-secure.

2 Models are unadjusted. Crude ORs are reported for obesity status (BMI ≥30 kg/m^2^).

3 Models adjusted for age, university, employment, race/ethnicity, meal plan, and marital status. Adjusted ORs are reported for obesity status.

To determine the differences in weight status and dietary variables by MP enrollment and food security status, 1-factor ANOVA was conducted across the following 4 groups: *1*) FS with an MP, *2*) FS without an MP, *3*) FI with an MP, and *4*) FI without an MP (**Table 5**). There was a statistically significant difference in BMI [*F* (3, 643) = 7.27, *P* < 0.001], WC [*F* (3, 642) = 5.64, *P* < 0.001], and HC [*F* (3, 645) = 5.05, *P* < 0.001]. A Tukey post hoc test revealed that FI students with MPs had a significantly higher BMI, WC, and HC than FS students with and without MPs. By the same token, a significantly higher proportion of FI students with MPs had obesity than of those without MPs (25.3% compared with 12.2%) [*χ*^2^ (2), 165) = 5.05, *P *= 0.025], whereas students who were both FS and lacking an MP had the lowest prevalence of obesity (4.6%). With respect to dietary intake, significant differences were found in the consumption of fruits and vegetables [*F* (3, 647) = 4.34, *P* = 0.005], sugars from SSBs [*F* (3, 643) = 15.16, *P* < 0.001], and total added sugars [*F* (3, 645) = 9.33, *P* < 0.001]. Specifically, FI students with MPs had a significantly lower intake of fruits and vegetables than FS students with MPs. However, there was no difference between FI students with MPs and those without MPs. FI students with MPs had the highest intake of added sugars and FS students without MPs had the lowest intake. No between-group differences were found in the consumption of dairy products, whole grains, calcium, or fiber.

**TABLE 5:** Comparisons of anthropometric measures and dietary intake by MP enrollment and food security status of second-year college students, USA, 2017^1^

Variable	FS with MP (a) (*n* = 316)	FS without MP (b) (*n* = 180)	FI with MP (c) (*n* = 85)	FI without MP (d) (*n* = 88)	F or *χ*^2^	Post hoc tests
Anthropometrics						
WC, cm	79.1 ± 0.5	77.3 ± 0.6	84.0 ± 1.6	83.0 ± 1.1	5.64**	c > a, b; d > b
HC, cm	100.9 ± 0.4	99.1 ± 0.6	103.5 ± 1.3	102.1 ± 1.0	5.05**	c > a, b; d > b
BMI, kg/m^2^	24.4 ± 0.2	23.7 ± 0.2	26.2 ± 0.7	25.3 ± 0.5	7.27***	c > a, b; d > b
Obese	29 (9.5)	8 (4.6)	21 (25.3)	10 (12.2)	26.01***	c > d; b < a, c, d
Not obese	276 (90.5)	165 (95.4)	62 (74.7)	72 (87.8)		
Dietary intake						
FV, CE/d	2.3 ± 0.04	2.0 ± 0.02	1.8 ± 0.07	1.9 ± 0.07	4.34**	a > c
Dairy, CE/d	1.4 ± 0.05	1.3 ± 0.05	1.4 ± 0.09	1.4 ± 0.08	0.77	—
Calcium, mg/d	853.1 ± 31.7	750.5 ± 26.6	791.4 ± 38.3	805.6 ± 43.6	1.85	—
Whole grains, oz/d	0.6 ± 0.04	0.5 ± 0.05	0.6 ± 0.08	0.8 ± 1.30	2.17	—
Fiber, g/d	13.5 ± 0.2	12.8 ± 0.3	12.6 ± 0.3	13.1 ± 0.4	1.92	—
Added sugars, tsp/d	12.1 ± 0.3	10.9 ± 0.3	14.8 ± 0.7	12.0 ± 0.4	9.33***	c > a, b, d; b < a, c, d
Sugar from SSBs, tsp/d	4.5 ± 0.25	3.51 ± 0.2	7.8 ± 0.8	5.1 ± 0.5	15.16***	c > a, b, d

^1^ *n* = 683. Values are means ± SEs or *n* (%) unless otherwise indicated. Df for all ANOVA numerators were 3 and for denominators ranged between 643 and 668, depending on missing data. **P < 0.01, ***P < 0.001. CE, cup-equivalent; FI, food-insecure; FS, food-secure; FV, fruits and vegetables; HC, hip circumference; MP, meal plan; SSB, sugar-sweetened beverage; WC, waist circumference.

## Discussion

The purposes of this study were to examine the sex-specific associations between food insecurity and obesity among college students and to compare the differences in dietary intake between FS and FI students. An exploratory aim of this study was to assess the differences in weight status and dietary intake by MP enrollment. Results suggested that college students are at risk of food insecurity and justify calls for interventions aimed to mitigate its burden. Twenty-five percent of students from this sample reported food insecurity. In addition, 22.3% were classified as marginally FS, displaying anxiety over food sufficiency. Consistently across both sexes, students with food insecurity were at higher risk of having obesity and poorer dietary intake than their counterparts. Although additional studies are needed to understand the observed associations, an exploration of weight status and dietary intake by MP enrollment showed a higher intake of added sugars and higher rates of obesity among FI students with MPs than among FI students without MPs and FS students regardless of MP status.

Food insecurity was positively related to obesity in a dose-response pattern even after adjusting for potential confounders. Compared with students who were highly FS, students with marginal food security and those with food insecurity were 3 and 5 times more likely to have obesity, respectively. Although “marginal food security” has been classified as “food security” in the US government's estimates, college students who are marginally FS may be more similar to FI students than to FS students in their sociodemographic characteristics, psychosocial profiles, and patterns of disease (27). Indeed, a growing body of evidence highlights the potential underestimation of the prevalence of poor health outcomes when marginally FS individuals are classified as FS (27). Because marginal food security status is characterized by feelings of uncertainty and anxiety, these emotional responses could mediate its association with obesity (28), highlighting the need to identify and target students with marginal food security status.

Contrary to previous studies of nonstudent adults reporting an association between food insecurity and obesity only among women (18, 29), food insecurity remained a significant predictor of obesity among both sexes after adjustment for demographic confounders. FI male and female students were 3 and 2 times more likely to have obesity than were their FS counterparts, respectively.

The mechanisms for the association between obesity and food insecurity are still not well elucidated. One proposed mechanism is that FI individuals may consume nutritionally inadequate diets, yet still have an energy intake that exceeds their daily requirements (30). Lacking sufficient financial resources or prioritizing obligatory expenses, college students may gravitate toward less expensive yet energy-dense foods and/or limit purchasing fresh produce (31). Indeed, results from the present study showed different food consumption patterns between FS and FI students. Compared with FS students, FI students had a higher intake of added sugars and lower fruit and vegetable consumption. These findings were in line with previous studies noting negative associations between food insecurity and dietary quality (32). Based on a 2015 systematic review by Darmon and Drewnowski (33) on the relation between food prices, diet quality, and diet costs in 151 studies, the energy density of the diet was inversely proportional to its cost, indicating that healthy dietary patterns that include fruits, vegetables, and whole grains are more expensive than those that include processed foods and refined grains. Thus, food insecurity may increase the consumption of energy-dense foods, which in turn may elevate the risk of weight gain and potential chronic diseases (34, 35).

This study identified the need for further exploration of MP subsidies as a mechanism to conjointly address food access and promote healthy food choices. The rate of obesity and added sugars intake were higher among FI students with MPs than among FI students lacking MPs. It is noteworthy, however, that MPs are defined differently on each campus varying from partial to all-you-can-eat, and these differences were not captured in the current study. FI students may alternate between cycles of scarcity and availability depending on the type of MP in which they are enrolled. From the current exploratory findings, it is plausible that FI students with access to the dining halls engage in an adaptive “buffering” behavior against any future uncertainty when MP benefits are running low. This observation is comparable with the pattern observed linking food assistance programs and obesity, in which the relation between these programs and obesity is greatest in those who are FI (18). The “food stamp cycle” hypothesis states that FI individuals may restrain from eating when resources are low but engage in overeating behaviors at the beginning of the month when food access is guaranteed (18). Nevertheless, findings showed that students without MPs had higher odds of food insecurity than their counterparts. Therefore, providing FI students with access to a campus MP may be an important aid to ensure that adequate food can be obtained consistently. It is also critical that future efforts consider opportunities that simultaneously support food security and obesity prevention.

Findings from the present study support theories suggesting the presence of underlying psychological and physiological processes that drive decisions toward energy-dense food choices and subsequent fat deposition in FI individuals (7). When comparing FS with FI students who were both enrolled in MPs, FI students displayed a significantly lower intake of fruits and vegetables. In addition, even though FI students with MPs had the highest added sugar intake and rates of obesity, FI students without MPs still exhibited higher rates than all other FS students. Recent hypotheses, such as the “insurance hypothesis” (7) and “resource scarcity hypothesis” (9), have focused on “adaptive strategies” as a more probative and mechanistic explanation for the association of obesity and food insecurity. The overarching premise of these hypotheses is that, under conditions of perceived food uncertainty, physiological and behavioral processes increase the deposition of body fat to buffer against any future food scarcity (7). Evidence for this relation was also observed in animal studies where subordinate animals that were exposed to experimental manipulation, or natural circumstances that threatened food security, increased body fat accretion (36) and obesogenic food intake (37), compared with more dominant animals. Alternatively, FI students may prefer energy-dense foods, which may be moderated by childhood experiences with food insecurity (38). Future research is needed to explore these potential mechanisms.

Interpretation of these findings necessitates consideration of study limitations. Although the study's cross-sectional nature allowed for the ascertainment of associations and development of hypotheses concerning relations between food insecurity and obesity, it is not possible to establish causality using this design. The use of longitudinal designs with repeated measures of obesity and food insecurity can provide an indication of causal pathways but experimental studies are necessary to establish causality.

Our findings may not apply to all US college students. Because the parent study was designed to include students at risk of poor health behaviors, the nature of this sample might have influenced our findings of the relation between food insecurity and obesity. Likewise, although our sample was drawn from 8 states, it was limited to those in the Eastern part of the United States, especially the Southeast. Other regions like the Midwest and West were not represented, and these tend to have lower proportions of food insecurity and obesity than others (39, 40). Thus, we cannot exclude the possibility of selection bias.

Although widely used in this population, the USDA AFSS has not been validated in college students. This factor may have introduced survey response error because it is unclear whether students interpret and respond to the questions like other populations. In addition, there have been suggestions that food insecurity experienced during childhood and adolescence has effects on weight gain later in life (38), and these earlier experiences were not captured in the study survey. Future studies should assess the history of food insecurity predating and including college, and examine how experiences with intermittent compared with chronic food insecurity affect weight status. Finally, we dichotomized students based on the presence or absence of an MP but the survey did not include questions pertaining to specific types of MPs. The absence of this question has limited our ability to examine the interaction of diet, food insecurity, and obesity by the different types of MPs available.

In conclusion, this study was the first to examine the sex-specific association of food insecurity with obesity among college students and explore the differences in obesity and dietary intake by MP enrollment. A relatively high prevalence of food insecurity was reported among this sample of college students. Furthermore, both male and female FI students had an elevated risk of obesity and tended to have less desirable dietary outcomes than their FS counterparts. Access to MPs may help improve food access, because students without MPs had greater odds of reporting food insecurity. On the other hand, FI students with MPs had the highest rates of obesity and added sugars intake. Subsidizing dining for students experiencing immediate food access issues and those at risk of food insecurity should be coupled with educational programming to improve the consumption of fruits and vegetables and reduce the intake of added sugars, specifically from SSBs. Continued efforts to implement and test the effectiveness of such strategies on college campuses are needed to reduce the burden of food insecurity and improve health in college students.

## Notes

Supported by USDA National Institute of Food and Agriculture award 2014-67001-21851 (to SEC).

Author disclosures: the authors report no conflicts of interest.

Abbreviations used: AFSS, Adult Food Security Survey; CE, cup-equivalent; FI, food insecure; FS, food secure; HC, hip circumference; MP, meal plan; NCI, National Cancer Institute; SSB, sugar-sweetened beverage; WC, waist circumference.
